# Applying ONCO-RADS to whole-body MRI cancer screening in a retrospective cohort of asymptomatic individuals

**DOI:** 10.1186/s40644-024-00665-z

**Published:** 2024-02-07

**Authors:** Yong-Sin Hu, Chia-An Wu, Dao-Chen Lin, Po-Wei Lin, Han-Jui Lee, Lo-Yi Lin, Chung-Jung Lin

**Affiliations:** 1 Department of Radiology, Taipei Hospital, Ministry of Health and Welfare, New Taipei, Taiwan; 2 School of Medicine, National Yang Ming Chiao Tung University, Taipei, Taiwan; 3 Department of Radiology, Taipei Veterans General Hospital, Taipei, Taiwan; 4 Department of Radiology, Shuang Ho Hospital, Taipei Medical University, Taipei, Taiwan; 5 Division of Endocrine and Metabolism, Department of Medicine, Taipei Veterans General Hospital, Taipei, Taiwan

**Keywords:** Whole-body magnetic resonance imaging, cancer screening, ONCO-RADS

## Abstract

**Background:**

Whole-body magnetic resonance imaging (WB-MRI) has emerged as a valuable tool for cancer detection. This study evaluated the prevalence rates of cancer in asymptomatic individuals undergoing WB-MRI according to the Oncologically Relevant Findings Reporting and Data System (ONCO-RADS) classifications in order to assess the reliability of the classification method.

**Methods:**

We retrospectively enrolled 2064 asymptomatic individuals who participated in a WB-MRI cancer screening program between 2017 and 2022. WB-MRI was acquired on a 3-T system with a standard protocol, including regional multisequence and gadolinium-based contrast agent-enhanced oncologic MRI. Results of further examinations, including additional imaging and histopathology examinations, performed at our institute were used to validate the WB-MRI findings. Two radiologists blinded to the clinical outcome classified the WB-MRI findings according to the ONCO–RADS categories as follows: 1 (normal), 2 (benign finding highly likely), 3 (benign finding likely), 4 (malignant finding likely), and 5 (malignant finding highly likely). Firth logistic regression analysis was performed to determine the associations between participant characteristics and findings of ONCO-RADS category ≥ 4.

**Results:**

Of the 2064 participants with median age of 55 years, 1120 (54.3%) were men, 43 (2.1%) had findings of ONCO-RADS category ≥ 4, and 24 (1.2%) had confirmed cancer. The cancer prevalence rates were 0.1%, 5.4%, 42.9%, and 75% for ONCO-RADS categories 2, 3, 4, and 5, respectively. In the multivariable model, older age (OR: 1.035, *p* = 0.029) and history of hypertension (OR: 2.051, *p* = 0.026), hepatitis B carrier (OR: 2.584, *p* = 0.013), or prior surgery (OR: 3.787, *p* < 0.001) were independently associated with the findings for ONCO-RADS category ≥ 4.

**Conclusions:**

The ONCO-RADS categories for cancer risk stratification were validated and found to be positively correlated with cancer risk. The application of ONCO-RADS facilitates risk-based management after WB-MRI for cancer screening.

**Supplementary Information:**

The online version contains supplementary material available at 10.1186/s40644-024-00665-z.

## Background

Whole-body magnetic resonance imaging (WB-MRI), which involves imaging of the head, neck, chest, abdomen, pelvis, and bones, has emerged as a valuable tool for cancer detection, particularly in the pediatric population with cancer predisposition syndrome. WB-MRI provides excellent soft tissue contrast resolution and is free from ionizing radiation [1, 2]. In a prospective observational study on patients with Li-Fraumeni syndrome, implementation of WB-MRI in the surveillance group provided early tumor detection and improved long-term survival (5 year overall survival rates, 88.8% vs. 59.6%), compared with non-surveillance group [3]. Recent advances in WB-MRI techniques have enabled imaging procedures to be completed within 60 to 90 min, depending on the sequence components [4–6]. The increasing popularity of WB-MRI as a screening tool for individuals who self-refer themselves for wellness checkups has resulted in the widespread utilization of the technique worldwide [5, 7]. However, a review of 12 studies revealed that abnormal findings requiring further evaluation were detected in up to 30% of 3331 asymptomatic individuals screened using WB-MRI [5]. A classification of these findings based on cancer risk is crucial to facilitate effective postscreening consultation and management.

In 2021, an international multidisciplinary expert panel proposed a set of guidelines, known as the Oncologically Relevant Findings Reporting and Data System (ONCO-RADS) guidelines, to establish a standardized approach for the acquisition and reporting of WB-MRI results for cancer screening [8]. Specifically, the ONCO-RADS guidelines employs a 5-category scoring system to evaluate abnormal findings based on the likelihood of cancer in multiple body regions. The standardized data collection method aims to reduce the variability of WB-MRI interpretations and direct risk-based management. However, the application of ONCO-RADS requires empirical validation. Thus, the present study assessed the reliability of ONCO-RADS and explored the prevalence rates of cancer in asymptomatic individuals undergoing WB-MRI according to ONCO-RADS classifications.

## Methods

### Participants and study design

The requirement for informed consent was waived because of the retrospective nature of this study. The STROBE reporting guidelines were implemented. From September 2017 to March 2022, 2203 self-referred individuals participated in a WB-MRI screening program at our institute. The WB-MRI screening program involves WB-MRI examinations, laboratory tests, and other optional imaging studies, including ultrasound and low-dose chest CT scan. Participants completed a questionnaire to collect data on current symptoms, personal medical history, surgical history, and family history of cancer. Individuals were included if they were aged ≥ 18 years, asymptomatic, and had been cancer-free for ≥ 5 years. Individuals with significant clinical symptoms (*n* = 21) or those who had not been cancer-free for ≥ 5 years (*n* = 118) were excluded. In total, 2064 participants were included for analysis.

### WB-MRI examinations and follow-up strategy

MRI was acquired on a 3-T system with a 70-cm bore (Magnetom Skyra; Siemens Healthcare, Erlangen, Germany). The sequence components for WB-MRI at our institute and the recommendation of ONCO-RADS are summarized in Table 1 [8]. In brief, T2-weighted images were acquired for all regions, with a slight difference in the imaging techniques according to the region. Regional multisequence oncologic MRI, including DWI, was performed for the brain, abdomen, and pelvis. After the administration of 0.1 mmol/kg gadolinium-based contrast agent (Gadovist, Shering, Berlin, Germany), dynamic T1 gradient echo images with fat saturation were acquired for the abdomen, followed by T1-weighted images for the other regions. The average empirical WB-MRI examination time was 68 min. No severe adverse reactions to the contrast agent were reported. Adjunct ultrasound examinations of the thyroid and whole abdomen were performed in all participants. Board-certified radiologists experienced in MRI interpretation reviewed all images and provided participants with a summary of the findings on site, along with a formal report in 7 days. Depending on the findings, specialty referrals for further investigations at our institute were arranged.

**Table 1:** Sequence components for WB-MRI at our institute and the recommendation of ONCO-RADS

Our institute			The recommendation of ONCO-RADS	
Region		Sequence description	Region	Sequence description
Brain		Axial 5-mm-thick T2 FLAIR with fat suppression (TR ms/TE ms/Matrix, 8000/92/320 × 224)	Brain	Axial 4–5-mm-chick T2 FLAIR
		Sagittal 1.5-mm-thick T1 MPRAGE with axial 1.5-mm-thick MPR (2000/2.49/256 × 256)	Whole -body	Axial 5-mm-thick T1 GRE with Dixon technique
		Coronal 5-mm-thick T2 TSE (4800/88/448 × 314)		Axial 5-mm-thick T2 TSE
		Axial 5-mm-thick DWI (b value = 0 and 1000 s/mm^2^) with corresponding ADC maps (5000/59/128 × 128)		Axial 5-mm-thick DWI (b value = 50–100 and 800–1000 s/mm^2^) with corresponding ADC maps, coronal MPR, and 3D MIP reconstruction (b value = 800–1000 s/mm^2^)
		Sagittal 1.5-mm-thick CE T1 MPRAGE with axial and coronal 1.5-mm-thick MPR (2000/2.49/256 × 256)		
Head and neck		Axial 3-mm-thick T2 TSE with fat suppression (5000/75/256 × 179)		
		Axial 3-mm-thick CE T1 GRE VIBE with fat suppression (3.78/1.55/320 × 240)		
Chest		Axial, coronal, and sagittal 6-mm-thick T2 HASTE (1000/98/320 × 182)	Chest	Axial < 3-mm-thick T1 GRE VIBE with short echo time
		Axial 3-mm-thick CE T1 GRE VIBE fat suppression with short echo time and coronal and sagittal 3-mm-thick MPR (1.63/3.53/320 × 208)		
Abdomen and pelvis		Coronal 4-mm-thick T2 HASTE (900/126/320 × 224)		
Abdomen		Axial 6-mm-thick T1 GRE with Dixon technique (150/1.23, 2.46/256 × 166)		
		Axial 6-mm-thick T1 TSE with fat suppression (200/1.23/256 × 154)		
		Axial 6-mm-thich heavily T2 TSE (900/166/320 × 208) with MR cholangiopancreatography (4500/735/284 × 269)		
		Axial 5-mm-thick DWI (b value = 0, 400, and 800 s/mm^2^) with corresponding ADC maps (6100/63/150 × 95)		
		Axial 3-mm-thick T1 GRE VIBE fat suppression with dynamic CE study and coronal 3-mm-thick MPR* (3.6/1.28/320 × 156)		
Pelvis	Male	Axial, sagittal, and coronal 3-mm-thick T2 TSE (4280/95/384 × 346)		
		Axial 3-mm-thick DWI (b value = 0, 1000, and 2000 s/mm^2^) with corresponding ADC maps (5600/75/120 × 120)		
	Female	Axial 4-mm-thick T1 TSE (230/2.46/320 × 224)		
		Axial 4-mm-thick (4000/97/384 × 346) and sagittal 3-mm-thick T2 TSE (4850/117/320 × 320)		
	Both	Axial 6-mm-thick DWI (b value = 400 s/mm^2^) (6100/63/150 × 95)		
		Axial 3-mm-thick CE T1 GRE VIBE with fat saturation (3.5/1.53/256 × 186)		
Spine		Sagittal 3-mm-thick T2 TSE (3000/93/320 × 256)	Spine	Sagittal 4–5-mm-thick T1 TSE
				Sagittal 4–5-mm-thick T2 with fat suppression or STIR

WB-MRI = whole-body magnetic resonance imaging; ONCO-RADS = Oncologically Relevant Findings Reporting and Data System; FLAIR = fluid-attenuated inversion recovery; TR = repetition time; TE = echo time; MPRAGE = magnetization-prepared rapid acquisition gradient echo; MPR = multiplanar reconstruction; GRE = gradient echo; TSE = turbo spin echo; DWI = diffusion-weighted imaging; ADC = apparent diffusion coefficient; 3D = three-dimensional; MIP = maximum intensity projection; CE = gadolinium-based contrast material enhanced; VIBE = volumetric interpolated breath-hold examination; HASTE = half-Fourier single-shot turbo spin echo; STIR = short inversion time inversion-recovery. * The dynamic CE study included arterial, portal venous, venous, and delayed phases; The coronal MPR included unenhanced and portal venous phases

### Study parameters and imaging evaluations

Participant demographic characteristics (age and sex), physical measurements (height, weight, blood pressure, and heart rate), medical history (hypertension, coronary artery disease, diabetes mellitus, hepatitis B, hepatitis C, and cured cancer), surgical history, family history of any cancer, WB-MRI results, and additional investigations for suspicious lesions were collected for analysis. We recorded the results of incidental lesions (benign lesions or cancer) that were validated by additional or follow-up imaging (ultrasound, CT, and dedicated MRI) and histopathology examinations performed in our institute. Two radiologists blinded to clinical outcomes who each had 17 and 8 years of experience in MRI interpretation independently evaluated the MRI images. The evaluators were asked to refer to the ONCO-RADS examples of the most frequently observed abnormal findings across multiple anatomic regions, including the head, neck, chest, abdomen, pelvis, and bones during interpretation. The suspicious findings for cancer were described using free text and were classified using the ONCO-RADS categories as follows: 1 (normal), 2 (benign finding highly likely), 3 (benign finding likely), 4 (malignant finding likely), and 5 (malignant finding highly likely) [8]. The consensus ONCO-RADS category was used for subsequent analysis. The highest ONCO-RADS category among the body regions was selected for per-participant analysis.

### Statistical analyses

The results are presented as medians (interquartile ranges) and numbers (percentages) for continuous and categorical variables, respectively. Interobserver agreement for the ONCO-RADS category between the two evaluators was measured using weighted kappa with linear weights. Cochran’s Q test was used to evaluate possible prevalence rate differences across the anatomic regions in the participants. Univariable and multivariable Firth logistic regression analyses with estimated odds ratios (ORs) were used to identify the participant characteristics associated with the WB-MRI findings for ONCO-RADS categories ≥ 4. Variables with *p* < 0.10 in univariable analyses were included in the multivariable analysis. Statistical analyses were performed using SPSS (version 22; SPSS, IBM, Dublin, Ireland). Statistical significance was set at *p* < 0.05.

## Results

### Participant characteristics, WB-MRI findings, and results of additional investigations

Participant characteristics are shown in Table 2. The median age of the participants at the time of WB-MRI was 55 years (45–63 years), and 1120 participants (54.3%) were men. The median body mass index of the participants was 23.9 kg/m^2^ (21.6–26.5 kg/m^2^). In total, 466 participants (22.6%) and 196 participants (9.5%) had a history of hypertension and hepatitis B carrier, respectively. Fifty-one (2.5%) participants had cancer cured for ≥ 5 years. Family history of cancer was reported in 863 participants (41.8%). In this study, 28 participants (1.4%) underwent additional imaging studies that included CT, and 7 participants (0.3%) underwent additional imaging studies that included dedicated MRI. Histopathology examinations were performed on 42 participants (2%) at a median interval of 36 days (19–55 days) after WB-MRI. Cancers were confirmed in 24 participants (1.2%).

**Table 2:** Characteristics of the participants, WB-MRI results, and additional investigations

	Value (Interquartile range)
Characteristic	Overall (*n* = 2064)
Median age (yr)	55 (45–63)
Male (%)	1120 (54.3)
Median measured height (cm)	165 (158.8–171.6)
Median measured weight (kg)	65.2 (56.5–75)
Median measured body mass index (kg/m^2^)	23.9 (21.6–26.5)
Median measured systolic blood pressure (mmHg)	121 (109–135)
Median measured diastolic blood pressure (mmHg)	76 (70–83)
Median measured heart rate (bpm)	71 (64–79)
Hypertension (%)	466 (22.6)
Coronary artery disease (%)	144 (7)
Diabetes mellitus (%)	185 (9)
Hepatitis B carrier (%)	196 (9.5)
Hepatitis C (%)	17 (0.8)
Cancer cured for ≥ 5 yrs (%)	51 (2.5)
Surgical history (%)	372 (18)
Family history of cancer (%)	863 (41.8)
**WB-MRI results** (%)	
ONCO-RADS category 1	0 (0)
ONCO-RADS category 2	1984 (96.1)
ONCO-RADS category 3	37 (1.8)
ONCO-RADS category 4	35 (1.7)
ONCO-RADS category 5	8 (0.4)
**Additional investigations (%)**	
CT (%)	28 (1.4)
Median interval between CT and WB-MRI (day)	1 (0–22)
Dedicated MRI (%)	7 (0.3)
Median interval between dedicated MRI and WB-MRI (day)	75 (32–1415)
Histopathology examination (%)	42 (2)
Median interval between histopathology and WB-MRI (day)	36 (19–55)
**Cancer**	24 (1.2)
Confirmed by imaging only	2 (0.1)
Confirmed by histopathology	22 (1.1)

WB-MRI = whole-body magnetic resonance imaging; ONCO-RADS = Oncologically Relevant Findings Reporting and Data System; CT = computed tomography

### Cancer detection based on ONCO-RADS categories

The kappa value for interobserver agreement for the ONCO-RADS category was 0.927 (95% confidence interval [CI]: 0.89–0.964). On a per-participant basis, 0 (0%), 1984 (96.1%), 37 (1.8%), 35 (1.7%), and 8 (0.4%) of 2064 participants had WB-MRI findings for the ONCO-RADS categories of 1, 2, 3, 4, and 5, respectively. The cancer prevalence rates were 0.1%, 5.4%, 42.9%, and 75% for ONCO-RADS categories 2, 3, 4, and 5, respectively.

The results of further investigation according to the body region and ONCO-RADS category are shown in Table 3. ONCO-RADS category ≥ 4 was observed in the head (*n* = 2, 0.1%), neck (*n* = 7, 0.3%), chest (*n* = 12, 0.6%), abdominal (*n* = 14, 0.7%), pelvic (*n* = 8, 0.4%), and bone (*n* = 1, < 0.1%) regions, without significant differences in prevalence rates among the regions (*p* = 0.355), except for the head and bone regions (*p* = 0.002). Cancers were confirmed in the head (*n* = 2, 0.1%), neck (*n* = 3, 0.1%), chest (*n* = 9, 0.4%), abdominal (*n* = 7, 0.3%), and pelvic (*n* = 4, 0.2%) regions, without significant differences in prevalence rates among these regions (*p* = 0.139). One participant had findings for ONCO-RADS category 4 in the chest and abdomen, which were confirmed as lung adenocarcinoma and renal urothelial carcinoma.

**Table 3:** Results of further investigation according to regions and ONCO-RADS categories

	Number/total number (%)						
Region and result	No investigation	Benign lesion			Cancer		
		Confirmed by imaging or follow-up	Confirmed by histopathology		Confirmed by further imaging	Confirmed by histopathology	
Head, total subjects	2046/2064 (99.1)	13/2064 (0.6)	3/2064 (0.1)		0		2/2064 (0.1)
ONCO-RADS 1–2	2046/2046 (100)	0	0		0		0
ONCO-RADS 3	0	13/16 (81.3)	3/16 (18.7)		0		0
ONCO-RADS 4	0	0	0		0		2/2 (100)
ONCO-RADS 5	0	0	0		0		0
Neck, total subjects	2050/2064 (99.3)	5/2064 (0.2)	6/2064 (0.3)		0		3/2064 (0.1)
ONCO-RADS 1–2	2048/2050 (99.9)	0	1/2050 (< 0.1)		0		1/2050 (< 0.1)
ONCO-RADS 3	0	3/7 (42.9)	4/7 (57.1)		0		0
ONCO-RADS 4	2/7 (28.6)	2/7 (28.6)	1/7 (14.3)		0		2/7 (28.6)
ONCO-RADS 5	0	0	0		0		0
Chest, total subjects	2041/2064 (98.9)	12/2064 (0.6)	2/2064 (0.1)		0		9/2064 (0.4)
ONCO-RADS 1–2	2040/2042 (99.9)	1/2042 (< 0.1)	0		0		1/2042 (< 0.1)
ONCO-RADS 3	0	8/10 (80)	1/10 (10)		0		1/10 (10)
ONCO-RADS 4	1/11 (9)	3/11 (27)	1/11 (9)		0		6/11 (55)
ONCO-RADS 5	0	0	0		0		1/1 (100)
Abdomen, total subjects	2053/2064 (99.5)	0	4/2064 (0.2)		2/2064 (0.1)		5/2064 (0.2)
ONCO-RADS 1–2	2047/2047 (100)	0	0		0		0
ONCO-RADS 3	2/3 (66.7)	0	1/3 (33.3)		0		0
ONCO-RADS 4	4/10 (40)	0	2/10 (20)		1/10 (10)		3/10 (30)
ONCO-RADS 5	0	0	1/4 (25)		1/4 (25)		2/4 (50)
Pelvis, total subjects	2057/2064 (99.7)	1/2064 (< 0.1)	2/2064 (0.1)		0		4/2064 (0.2)
ONCO-RADS 1–2	2052/2052 (100)	0	0		0		0
ONCO-RADS 3	2/4 (50)	1/4 (25)	1/4 (25)		0		0
ONCO-RADS 4	2/5 (40)	0	1/5 (20)		0		2/5 (40)
ONCO-RADS 5	1/3 (33.3)	0	0		0		2/3 (66.7)
Bones, total subjects	2062/2064 (99.9)	2/2064 (0.1)	0		0		0
ONCO-RADS 1–2	2062/2062 (100)	0	0		0		0
ONCO-RADS 3	0	1/1 (100)	0		0		0
ONCO-RADS 4	0	1/1 (100)	0		0		0
ONCO-RADS 5	0	0	0		0		0

ONCO-RADS = Oncologically Relevant Findings Reporting and Data System

**Fig. 1:**
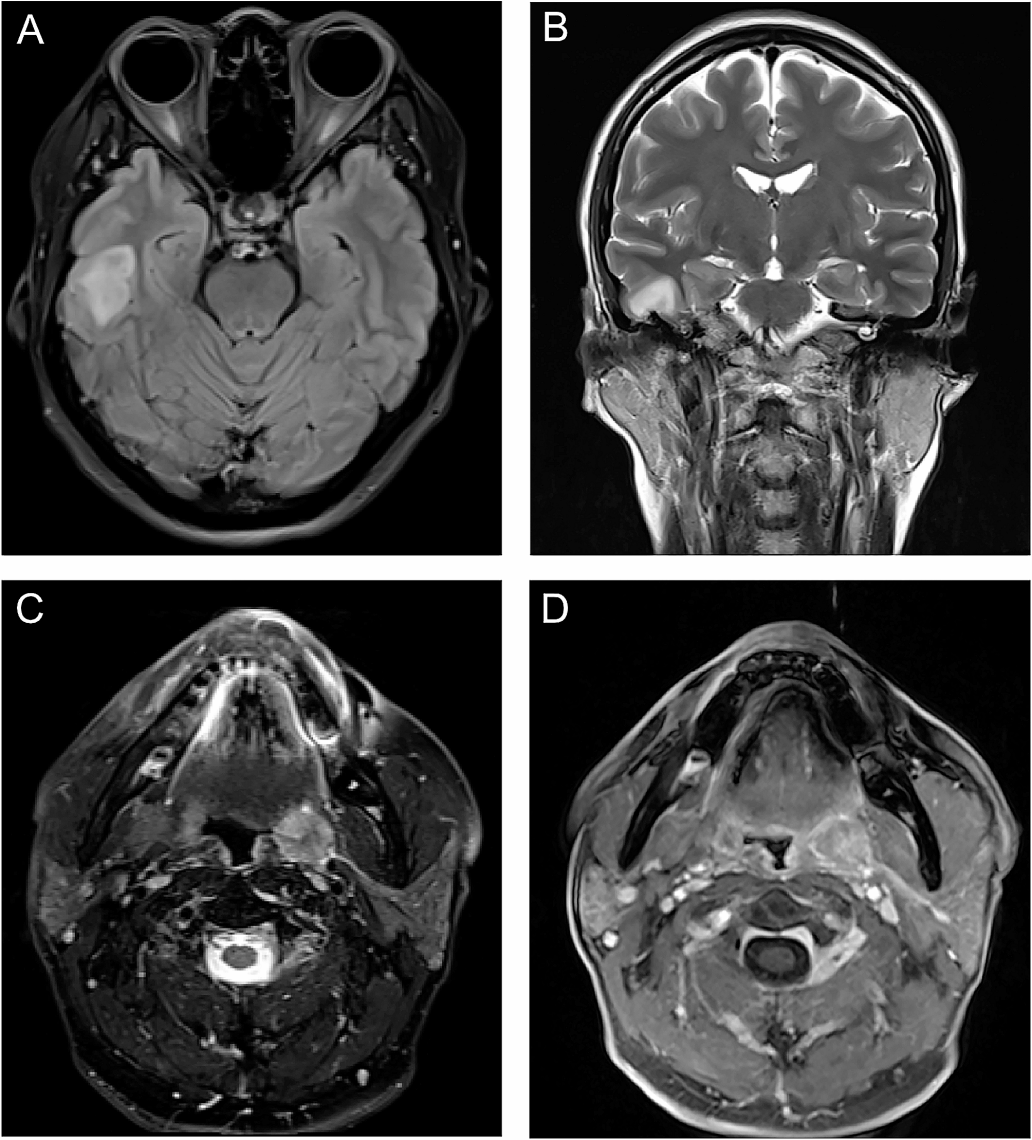
Imaging findings with Oncologically Relevant Findings Reporting and Data System category 4 in the head region. Axial T2 fluid-attenuated inversion recovery **(A)** and coronal T2-weighted **(B)** images show a hyperintense area in right temporal lobe of 41-year-old woman. Lesion was confirmed to be anaplastic astrocytoma after surgical resection. In 61-year-old man, axial T2-weighted **(C)** and contrast-enhanced T1-weighted **(D)** images reveal left tonsil lesion with heterogeneously high signal intensity, which was diagnosed as squamous cell carcinoma after biopsy

**Fig. 2:**
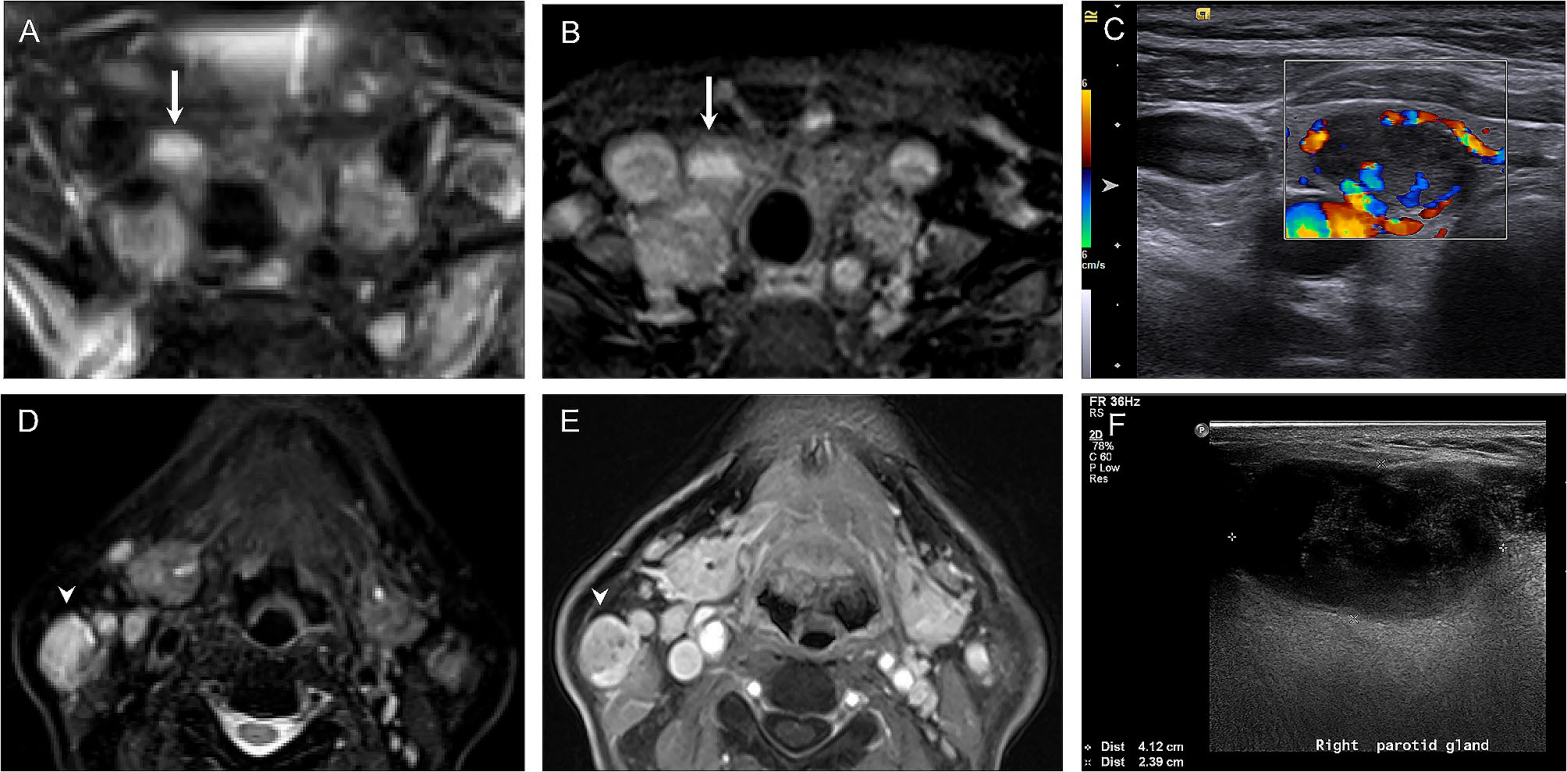
Imaging findings with Oncologically Relevant Findings Reporting and Data System category 4 in neck region. Axial T2-weighted **(A)** and contrast-enhanced T1-weighted **(B)** images show solid thyroid nodule (arrows) in right lobe of 63-year-old woman. Corresponding color Doppler ultrasound image **(C)** reveals 1.3-cm hypoechoic and hypervascular lesion with histopathology diagnosis of papillary carcinoma. In 67-year-old man, solid lesion (arrowheads) with adjacent daughter nodule was detected in right parotid gland, demonstrating hyperintensity on T2-weighted image **(D)** and contrast enhancement on T1-weighted image **(E)**. Lesion was hypoechoic on ultrasound image **(F)** and was determined to be Warthin’s tumor by fine-needle aspiration cytology

The number of incidental lesions confirmed by further investigations is summarized in Table 4. In the head region, all 16 lesions with ONCO-RADS category 3 were confirmed to be benign, and 2 lesions with ONCO-RADS category 4 were malignant (Fig. 1). All participants received neck ultrasound as a part of the cancer screening program, and one histopathology-confirmed 7-mm papillary thyroid carcinoma was not visible on MRI. All 7 neck lesions with ONCO-RADS category 3 were benign. Of the 7 ONCO-RADS-category-4 neck lesions, 2 did not undergo further investigation, 2 (29%) were papillary thyroid carcinoma (Fig. 2A-C), and 3 were benign, of which 2 were salivary gland tumors (Fig. 2D-F) and 1 was a thyroid nodule.

**Table 4:** Number of incidental lesions confirmed by further investigations

Region	Benign lesions (*n* = 50)	Cancers (*n* = 25)	
		Type	Stage
Head	Meningioma (8)	Anaplastic astrocytoma (1)	Grade 3 (1)
	Pituitary adenoma (4) Vestibular schwannoma (4)	Tonsil squamous cell carcinoma (1)	Stage IVa (1)
Neck	Thyroid nodule (8) Granulomatous thyroiditis (1) Warthin’s tumor (1) Parotid gland tumor (1)	Papillary thyroid carcinoma (3)	Stage I (2) and II (1)
Chest	Pneumonia (5)	Lung adenocarcinoma (7)	Stage IA1 (4), IA2 (1), IA3 (1), and IVA (1)
	Lung inflammatory nodule (4) Lung nodule (3) Castleman disease (1) Ectopic thyroid goiter (1)	Thymoma (2)	Stage I (2)
Abdomen	Gastric inflammatory polyp (1)	Renal cell carcinoma (3)	Stage I (3)
	Adrenal myelolipoma with hemorrhage (1) Renal oncocytoma (1) Schwannoma of the psoas (1)	Gastrointestinal stromal tumor of the stomach (2) Renal urothelial carcinoma (1) Hepatocellular carcinoma (1)	Stage IA (1) and IB (1) Stage I (1) Stage II (1)
Pelvis	Endometrial polyp (2) Paratubal cyst (1)	Prostatic adenocarcinoma (4)	Stage IIC (2) and IIIB (2)
Bones	Spinal meningioma (1) Paraspinal neurogenic tumor (1)		

**Fig. 3:**
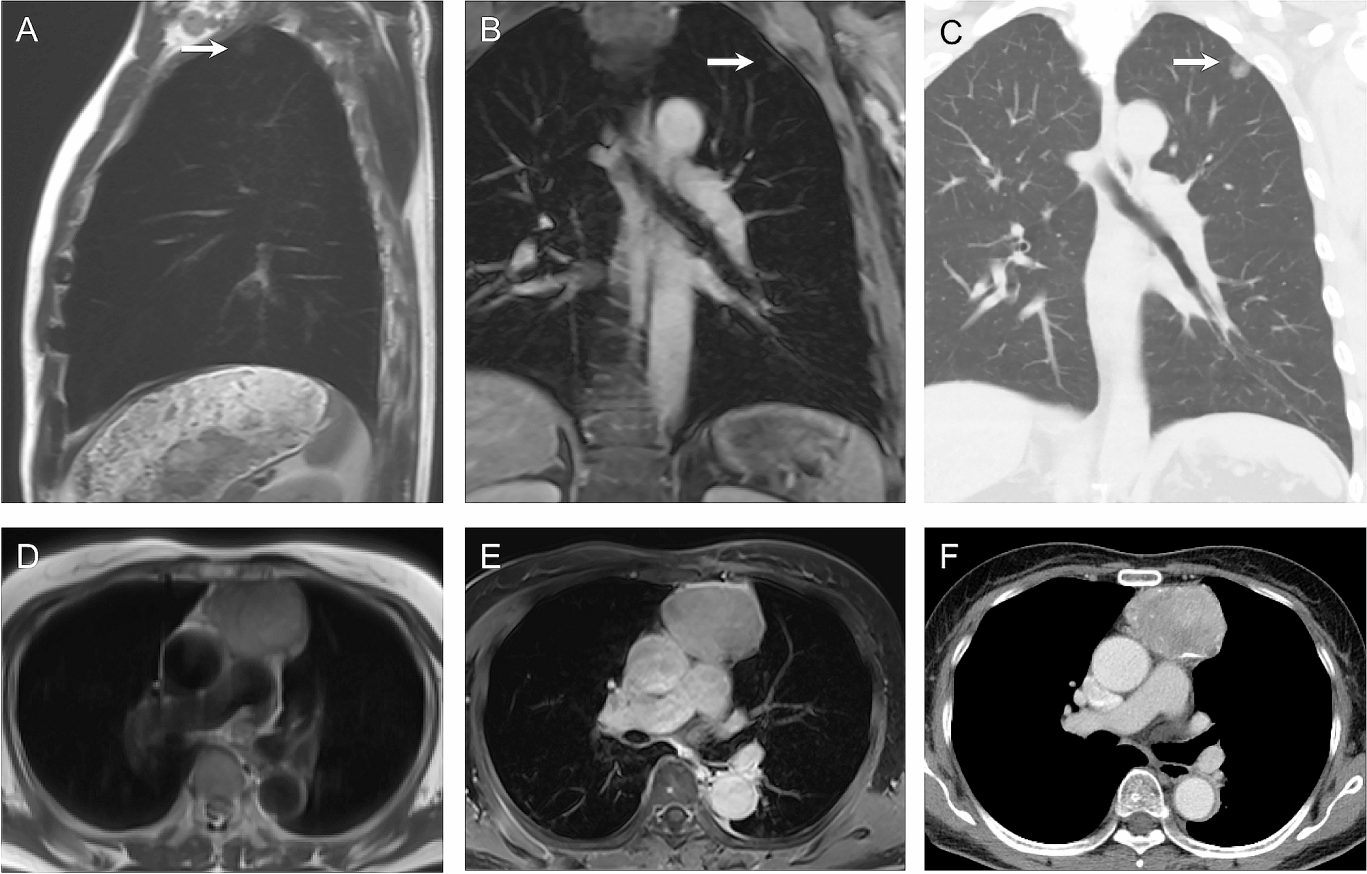
Imaging findings with Oncologically Relevant Findings Reporting and Data System category 4 in chest region. Sagittal T2 half-Fourier single-shot turbo spin echo (HASTE) **(A)** and coronal contrast-enhanced T1 gradient echo (GRE) **(B)** images reveal 17-mm subpleural nodule (arrows) in upper lobe of left lung in 62-year-old man. Lesion is seen on corresponding coronal CT scan lung window **(C)** and was confirmed as lung adenocarcinoma after surgical resection. In 69-year-old woman, 5.8-cm anterior mediastinal mass with hyperintensity was noted on T2 HASTE image **(D)** and contrast enhancement on T1 GRE image **(E)**. Corresponding axial CT scan **(F)** demonstrates calcification in mass and thymoma was diagnosed after surgical resection

Of 2064 participants, 785 (38%) underwent chest CT within a 30-day interval from WB-MRI. One 9-mm pure ground-glass lung nodule undetected by MRI was proven to be adenocarcinoma. Of 10 ONCO-RADS-category-3 lesions in the chest region, 9 (90%) were benign. One ONCO-RADS-category-3 lung lesion, considered to be inflammatory consolidation on MRI, appeared as a 23-mm part-solid ground-glass nodule on CT and was confirmed to be malignant. Of the 12 chest lesions with ONCO-RADS category ≥ 4, 5 were lung adenocarcinomas and 2 were thymomas (Fig. 3).

**Fig. 4:**
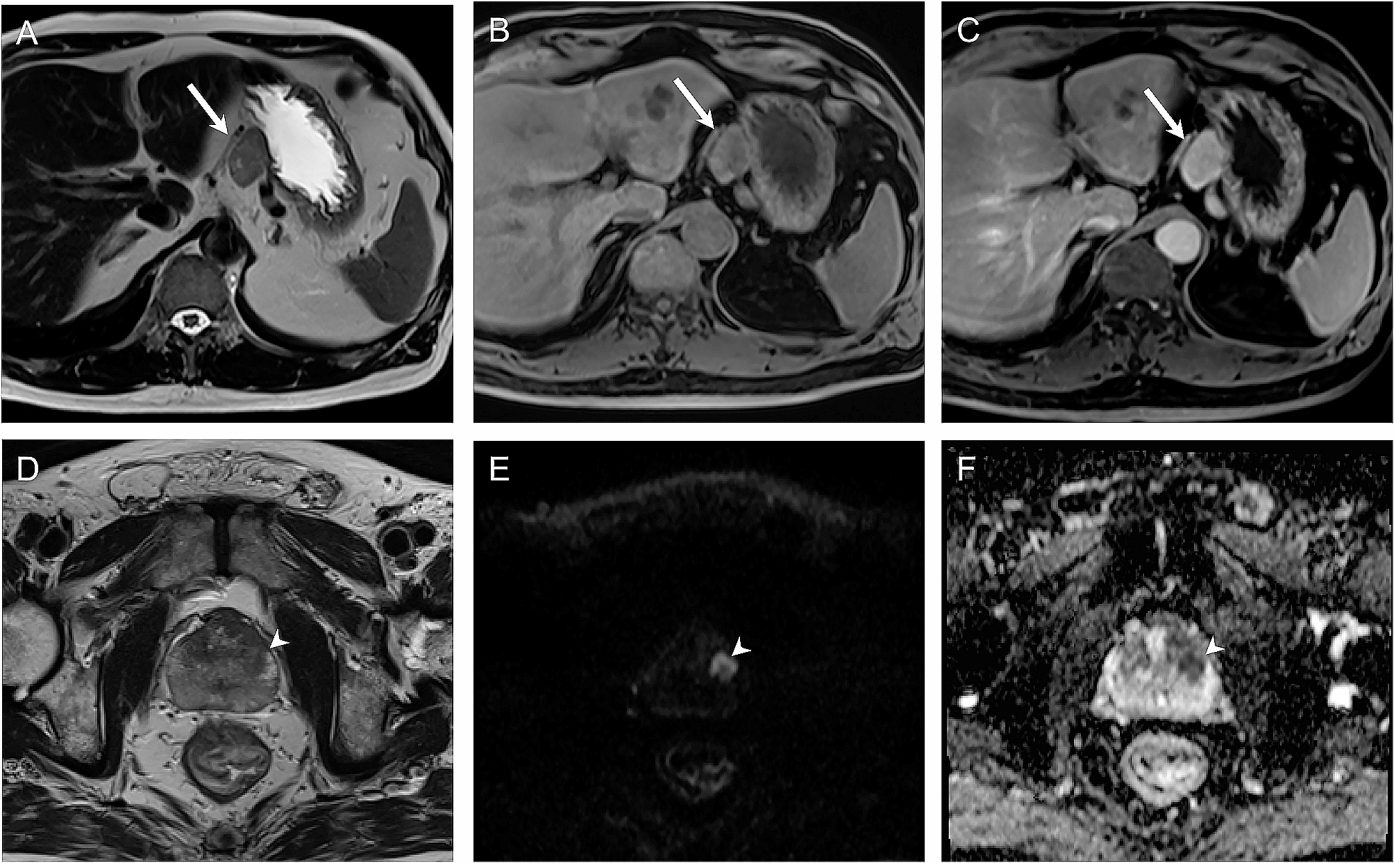
Imaging findings with Oncologically Relevant Findings Reporting and Data System (ONCO-RADS) category 4 in abdominal region and ONCO-RADS category 5 in pelvic region. In 75-year-old man, 3.6-cm exophytic mass (arrows) from lesser curvature of stomach is depicted on axial T2-weighted image **(A)** and T1-weighted images before **(B)** and after **(C)** contrast enhancement. Diagnosis of stromal cell tumor was given after laparoscopic resection. Axial T2-weighted image **(D)** revealed focal lesion (arrowheads) in left transition zone with low signal intensity, measuring more than 1.5 cm. Lesion shows marked diffusion restriction on diffusion-weighted imaging **(E)** and corresponding apparent diffusion coefficient map **(F)** and was classified as ONCO-RADS category 5. Prostate cancer was confirmed in 58-year-old man after biopsy directed by cognitive MRI-transrectal-ultrasound fusion technique

In the abdominal region, 4 ONCO-RADS-category-4 lesions that did not undergo further examination included two 3-cm suprarenal lesions, one 4-cm pancreatic cystic lesion, and one 8-mm gastric submucosal lesion. Of the 14 abdominal lesions with ONCO-RADS category ≥ 4, 7 (50%) were confirmed as cancers (Fig. 4A-C). Of the 8 pelvic lesions with ONCO-RADS category ≥ 4, 4 (50%) were prostatic adenocarcinoma (Fig. 4D-F), 1 was endometrial polyp, and 3 were prostatic lesions that did not undergo further examination. In the bone region, one ONCO-RADS-category-3 lesion was spinal meningioma, and one ONCO-RADS-category-4 lesion was a paraspinal neurogenic tumor.

### Factors associated with WB-MRI findings for ONCO-RADS category ≥ 4

The results of univariable and multivariable Firth logistic regression analyses of the associations between participant characteristics and WB-MRI findings for ONCO-RADS category ≥ 4 are presented in Table 5. In the multivariable model, older age (OR: 1.035, 95% CI: 1.004–1.068, *p* = 0.029), history of hypertension (OR: 2.051, 95% CI: 1.086–3.868, *p* = 026), history of hepatitis B carrier (OR: 2.584, 95% CI: 1.222–5.467, *p* = 0.013), and prior surgery (OR: 3.787, 95% CI: 1.992–7.197, *p* < 0.001) were independently associated with the WB-MRI findings of ONCO-RADS category ≥ 4.

**Table 5:** Univariable and multivariable Firth logistic regression analyses for factors associated with ONCO-RADS ≥ 4

Variable	No. of cases	Univariable		Multivariable	
		OR (95% CI)	*p* Value	OR (95% CI)	*p* Value
Age, yr	2064	1.056 (1.028–1.086)	< 0.001	1.035 (1.004–1.068)	0.029 *
Male	1120	0.9666 (0.531–1.757)	0.909		
Height, cm	2064	0.982 (0.954–1.01)	0.212		
Weight, kg	2064	1.001 (1–1.003)	0.067		
Body mass index, kg/m^2^	2064	1.004 (1–1.008)	0.06	1.003 (0.999–1.007)	0.163
Systolic blood pressure, mmHg	2064	1.007 (0.993–1.023)	0.331		
Diastolic blood pressure, mmHg	2064	1.005 (0.975–1.035)	0.767		
Heart rate, bpm	2064	1.004 (0.999–1.009)	0.131		
Hypertension	466	3.078 (1.687–5.618)	< 0.001	2.051 (1.086–3.868)	0.026 *
Coronary artery disease	144	1.148 (0.38–3.468)	0.806		
Diabetes mellitus	185	1.457 (0.589–3.608)	0.415		
Hepatitis B carrier	196	2.694 (1.294–5.611)	0.008	2.584 (1.222–5.467)	0.013 *
Hepatitis C	17	4.29 (0.785–23.455)	0.093	2.303 (0.4–13.247)	0.35
Cancer cured for ≥ 5 yrs	51	3.516 (1.138–10.87)	0.029	1.162 (0.35–3.855)	0.806
Surgical history	372	4.991 (2.733–9.114)	< 0.001	3.787 (1.992–7.197)	< 0.001 *
Family history of cancer	863	1.219 (0.67–2.218)	0.517		

ONCO-RADS = Oncologically Relevant Findings Reporting and Data System; OR = odds ratio

* Statistical significance

## Discussion

Nearly 30% of scanned participants have potentially relevant findings according to WB-MRI [5, 9]. Classification of abnormal findings based on cancer risk is essential to reduce unnecessary fear and to avoid additional investigations that have the potential to cause harm [10]. In prior studies, examinations were primarily performed using 1.5-T scanners, and the findings were interpreted based on heterogeneous classification systems with 2 to 5 categories [6, 9, 11, 12]. In the present study, the WB-MRI results of a relatively large sample of 2064 asymptomatic participants were presented as ONCO-RADS categories. In addition, the same sequence components were consistently performed on a 3-T scanner. The WB-MRI findings for ONCO-RADS category ≥ 4 and confirmed cancers were reported in 2.1% and 1.2% of the participants, respectively. Similarly, a review of 12 studies revealed that the prevalence rates of WB-MRI findings of suspected cancers and subsequently confirmed cancers were 1.8% and 1.1%, respectively [5]. 

Studies have suggested that the numbers of incidental WB-MRI findings increase with age [6, 13, 14]. In 229 participants undergoing WB-MRI for a routine health checkup, those aged ≥ 52 years were more likely to have incidental lesions than those aged < 52 years [13]. By including 666 WB-MRIs performed in individuals in the general population, Cieszanowski et al. [6] observed that incidental lesions requiring medical attention were more prevalent among individuals aged > 50 years. In the present study, the independent factors associated with the findings of ONCO-RADS category ≥ 4 were increased age, history of hypertension, hepatitis B carrier, and prior surgery. Causal relationships between these factors and certain cancers are difficult to ascertain given the low prevalence of cancer in asymptomatic individuals. We postulate that these factors reflect the individual health status associated with abnormal WB-MRI findings. By contrast, history of treated cancer and family history of cancer were not associated with abnormal WB-MRI findings. Family history of cancer was observed in nearly 25% of the participants in a study [15]. Although we did not examine the cancer types in detail, 41.8% of our participants had a family history of cancer, which may have motivated them to participate in the WB-MRI health checkup program. Nevertheless, individual pertinent histories should be collected before WB-MRI for personalized cancer risk stratification [8]. 

According to the analysis of findings for ONCO-RADS category ≥ 4 across body regions, the prevalence rates were 0.4% for the head and neck, 0.6% for the chest, and 1.1% for the abdomen and pelvis. Only one finding (< 0.1%) for ONCO-RADS category > 4 was observed in the bone. In a meta-analysis, the pooled prevalence rates of suspicious findings for cancer were 0.6% on brain MRI, 0.6% on thorax MRI, and 1.3% on abdominal MRI [16]. In the present study, approximately half of the findings for ONCO-RADS-category ≥ 4 were confirmed to be cancers, including 0.2% in the head and neck, 0.3% in the chest, and 0.5% in the abdomen and pelvis. Neck ultrasound and chest CT were supplementary examinations in our cancer screening program. These examinations helped identify 3 false-negative cases that had been classified as ONCO-RADS category ≤ 3. Specifically, 1 thyroid cancer and 2 lung adenocarcinomas were detected through these examinations. In addition, 7 benign lesions in the neck and chest had been classified as ONCO-RADS category 4 (malignant finding likely), comprising 1 thyroid follicular nodule, 2 salivary gland solid tumors, 3 lung nodules > 8 mm, and 1 Castleman disease with mediastinal masses. The aforementioned false findings corroborated with the examples provided by the ONCO-RADS guidelines and highlight the inferior resolution of MRI compared with ultrasound and chest CT in the assessment of thyroid and lung lesions [13, 17, 18]. These limitations of WB-MRI should be acknowledged, and additional evaluations should be made available according to the established guidelines for the management of incidental findings in asymptomatic individuals [19, 20]. 

WB-MRI is increasingly performed for asymptomatic individuals desiring to live a longer and heathier life in an attempt to detect tumors for timely interventions before symptomatic manifestation [5, 7]. This study provided a real-world overview of WB-MRI results of self-referred individuals according to the ONCO-RADS categories. After WB-MRI, several of the participants underwent surgical resection for early-stage cancer or benign tumors, namely anaplastic astrocytoma, pituitary macroadenoma, lung cancer, gastrointestinal stromal tumor, renal cancer, and prostate cancer, which potentially extended their life expectancy or improved their quality of life. Moreover, approximately 96% of the participants were classified as having ONCO-RADS category 2 (benign findings highly likely) and did not need to undergo further examinations. By using a retrospective cohort that underwent WB-MRI for cancer screening, we showed that the ONCO-RADS categories may enhance communication between consultants and participants and facilitate a management strategy based on cancer risk.

Our study has several limitations. First, this was a single-institute retrospective study in asymptomatic individuals who underwent an out-of-pocket WB-MRI screening program, and the results are subject to selection bias of individuals with higher socioeconomic status and are thus not generalizable to the general population. Second, 10 of 43 participants (23%) with findings of ONCO-RADS categories ≥ 4 did not have further confirmation, which may underestimate the confirmed cancer rate. However, this limitation represents the actual clinical situation and is present in similar studies [6, 11, 14]. Third, the sequence components in this study were not identical to those recommended by the ONCO-RADS guidelines [8]. Limbs were not included in WB-MRI because the prevalence of suspicious tumors in limbs in the general population is low [5]. We used a contrast agent for the dynamic contrast-enhanced imaging of the abdomen to detect hepatocellular carcinoma, which is one of the most common cancer types in Asia [21]. Contrast-enhanced T1-weighted imaging of other body regions was subsequently performed to substitute for DWI of the neck and chest because of the increased susceptibility effects in DWI with 3-T scanners [8]. Nevertheless, the use of a contrast agent is considered an invasive imaging approach and is therefore not recommended in the ONCO-RADS guidelines [8]. Further research is required to examine the value of a contrast agent in improving the diagnostic performance of WB-MRI in asymptomatic individuals. Finally, it is unclear whether the benefits of health improvement from early cancer detection and timely intervention in a small percentage of individuals outweigh the overall cost and risk of potential harm from additional investigations after WB-MRI. The cost-effectiveness of WB-MRI cancer screening in the general population needs to be validated in long-term prospective clinical trials.

## Conclusions

The ONCO-RADS guidelines are designed to classify abnormal WB-MRI findings possibly indicating malignant tumors. In this retrospective cohort of 2064 asymptomatic individuals undergoing WB-MRI for cancer screening, 43 individuals (2.1%) had findings of ONCO-RADS category ≥ 4, and 24 (1.2%) had cancers confirmed by subsequent examinations. We validated that ONCO-RADS classification is positively correlated with cancer risk. Individuals with older age or a history of hypertension, hepatitis B carrier, or prior surgery were more likely to have findings for ONCO-RADS-category ≥ 4. Incorporation of neck ultrasound and low-dose chest CT may improve cancer detection performance in WB-MRI screening programs.

### Electronic supplementary material

Below is the link to the electronic supplementary material.

Supplementary Material 1

## Data Availability

Not applicable.

## Abbreviations

WB-MRI: whole-body magnetic resonance imaging
ONCO-RADS: Oncologically Relevant Findings Reporting and Data System
ORs: odds ratios
CI: confidence interval
